# A qualitative synthesis of the positive and negative impacts related to delivery of peer-based health interventions in prison settings

**DOI:** 10.1186/s12913-016-1753-3

**Published:** 2016-09-29

**Authors:** Jane South, James Woodall, Karina Kinsella, Anne-Marie Bagnall

**Affiliations:** 1 Centre for Health Promotion Research, School of Health and Community Studies, Leeds Beckett University, CL 505, Calverley Building, City Campus, Leeds, LS1 3HE UK; 2 Centre for Health Promotion Research, School of Health and Community Studies, Leeds Beckett University, Leeds, UK

**Keywords:** Offender health, Systematic review, Qualitative research, Lay involvement, Peer support, Peer education, Health promotion, Implementation

## Abstract

**Background:**

Peer interventions involving prisoners in delivering peer education and peer support in a prison setting can address health need and add capacity for health services operating in this setting. This paper reports on a qualitative synthesis conducted as part of a systematic review of prison-based peer interventions. One of the review questions aimed to investigate the positive and negative impacts of delivering peer interventions within prison settings. This covered organisational and process issues relating to peer interventions, including prisoner and staff views.

**Method*s*:**

A qualitative synthesis of qualitative and mixed method studies was undertaken. The overall study design comprised a systematic review involving searching, study selection, data extraction and validity assessment. Studies reporting interventions with prisoners or ex-prisoners delivering education or support to prisoners resident in any type of prison or young offender institution, all ages, male and female, were included. A thematic synthesis was undertaken with a subset of studies reporting qualitative data (*n* = 33). This involved free coding of text reporting qualitative findings to develop a set of codes, which were then grouped into thematic categories and mapped back to the review question.

**Results:**

Themes on process issues and wider impacts were grouped into four thematic categories: peer recruitment training and support; organisational support; prisoner relationships; prison life. There was consistent qualitative evidence on the need for organisational support within the prison to ensure smooth implementation and on managing security risks when prisoners were involved in service delivery. A suite of factors affecting the delivery of peer interventions and the wider organisation of prison life were identified. Alongside reported benefits of peer delivery, some reasons for non-utilisation of services by other prisoners were found. There was weak qualitative evidence on wider impacts on the prison system, including better communication between staff and prisoners. Gaps in evidence were identified.

**Conclusions:**

The quality of included studies limited the strength of the conclusions. The main conclusion is that peer interventions cannot be seen as independent of prison life and health services need to work in partnership with prison services to deliver peer interventions. More research is needed on long-term impacts.

**Systematic review registration:**

PROSPERO ref: CRD42012002349.

**Electronic supplementary material:**

The online version of this article (doi:10.1186/s12913-016-1753-3) contains supplementary material, which is available to authorized users.

## Background

Prison is recognised by the World Health Organization (WHO) as an important setting for health because of the opportunities to improve the health of an at-risk population and address the major health inequalities that exist [1]. Health services operating within the criminal justice system have duties to meet prisoners’ rights to healthcare equivalent to that received by the wider population [2, 3]. In England and Wales, offender health services are the joint responsibility of the National Offender Management Service, Public Health England (the national public health agency) and the NHS [3]. Whilst the health challenges are significant [1], there are also organisational challenges in a social context where security concerns dominate and there may be resistance to professional help [4]. In this difficult environment, peer interventions involving prisoners in service delivery may offer a means to address health need and add service capacity [5, 6]. Health gains within prison may also have wider effects post-release, including reduced recidivism [7].

Peer interventions are an established feature in prison systems in many countries, with great diversity in terms of type of prison and intervention focus [8–10]. Peer workers, here defined as prisoners or ex-prisoners who deliver peer education or peer support in a voluntary or paid capacity in the prison, can act as mediators between professional services and prisoners. In England and Wales, peer interventions are provided by health services and also by third sector organisations, the prison service, and other education and welfare services, with a variety of schemes in operation [8] (see Table 1). For example, the Listener scheme, widely available across adult prisons as part of suicide prevention, involves trained prisoners providing a ‘listening ear’ for prisoners in distress [10].

**Table 1:** Examples of peer schemes operating in prisons in England and Wales

Prison Listeners	peer support to alleviate prisoner distress and prevent suicide
Insiders	peer support delivered in reception and first night suites
Health trainers	peer advice and support on healthy lifestyles and mental health
Toe-by-Toe	a peer-based reading scheme to improve prisoner literacy
Health care representatives	improving access to health services and service delivery
Peer mentoring	provision of positive role models, often for benefit of younger offenders
Prisoner Information Desk (PID) workers	signposting to sources of information and support
Peer advisors	focused on supporting resettlement, housing and employment

There is a need for robust evidence of effectiveness at the individual level, that is identifying the outcomes for prisoners who use peer interventions; however there is also a need to understand wider impacts if health services are to work well and be sustainable. It is suggested that having a lay workforce positively affects prison life and reduces demand for services [10]. The dominant nature of the setting means that peer interventions can affect the determinants of prison health and also be affected by factors within that setting. The aim of this paper is to report on a qualitative synthesis on positive and negative impacts relating to the delivery of peer interventions that was conducted as part of a systematic review of prison-based peer interventions [11].

### Study aims

The primary aim of the study was to review the effectiveness and cost-effectiveness of peer-based interventions in prison settings (results are reported elsewhere [11, 12]). A secondary aim was to provide research-based information on types of intervention, costs and benefits to aid decision making within prison health services. The conceptual framework incorporated the determinants of offender health across the life course, the prison as a unique setting and peer interventions as potential mechanisms for health behaviour change or risk reduction. This was represented as a preliminary logic model [13], which mapped assumed links between context, interventions, mechanisms, individual-level and organisational outcomes. There were four review questions; three of these concerned the traditional assessment of effectiveness by examining the effects of peer-based interventions on prisoner health, comparison between peer and professional interventions, and cost effectiveness (results reported elsewhere [12]). This paper concerns the fourth review question (Review Question 2) which aimed to investigate the positive and negative impacts of delivering peer-based interventions on health services within prison settings. This review question concerned organisational and process issues, including prisoner views on peer delivery. It was anticipated that mainly qualitative evidence from process evaluations or studies reporting qualitative interview findings would be included for this question.

## Methods

### Study design

The study design was a mixed method systematic review involving traditional stages of searching, study selection, data extraction and validity assessment [11]. A full systematic review protocol detailing search strategies and review methods was developed and published on PROSPERO (ref: CRD42012002349). This paper focuses on qualitative synthesis methods, but first a brief overview of the review process is given.

### Search strategies

A range of 19 electronic databases were searched for publications since 1985, including those reporting clinical or health service research, e.g. MEDLINE, CINAHL; and social science research e.g. Sociological Abstracts, Campbell Collaboration Database. Strategies to identify relevant grey literature included scanning conference abstracts, website searches and requests to organisations related to offender health [11]. An expert symposium held in 2012 helped gather specialist knowledge [14] and a number of UK publications were identified through this route.

### Study selection

Inclusion criteria were drawn up based on the PICOS framework [15]. The population was prisoners resident in prisons and Young Offender Institutions in any country, all ages, male and female. Peer-based interventions were defined as having prisoners or ex-prisoners delivering interventions to prisoners. Outcomes had to relate to prisoner health and determinants of health, process outcomes or views of prison populations. Study designs included quantitative, qualitative and mixed method evaluations. Two reviewers independently screened abstracts and then selected studies, with disagreements resolved initially by discussion between the researchers in relation to the inclusion/exclusion criteria. Where further clarification was needed, the study was discussed by the whole team and decisions were recorded.

### Data extraction

Data were extracted across fields including: population; setting/type of institution; health or social issue; delivery method; outcomes. Studies reporting qualitative data were assessed using the EPPI Centre framework for validity of qualitative research [16]. Quantitative and qualitative data from included studies were then synthesised separately using appropriate methods for each type of data. The final stage involved combining results for each review question into a narrative account [17].

### Qualitative synthesis

A thematic synthesis of included studies reporting qualitative data (*n* = 33) was undertaken using methods described by Thomas and Harden [18]. This method was chosen because the quality of reporting of qualitative results and the lack of thick descriptions in most included papers meant that meta-ethnography was unsuitable. An inductive approach to coding was used in preference to a pre-determined framework in order to capture the full range of impacts within the prison system. Familiarisation with a sample of papers preceded the development of an initial coding framework agreed by all qualitative review team members [JW, KK, AMB, JS]. For each study, the abstract and any sections of the publication reporting qualitative findings were included in the thematic analysis, as described by Thomas and Harden [18]. Two reviewers [JW, KK] worked independently to free code textual data (both reports of qualitative findings and verbatim quotations from interview data), adding new codes as required until a complete set of descriptive codes was obtained. The next stage involved grouping the descriptive codes (*n* = 99) into organising codes and finally into thematic categories using an iterative process to obtain the best fit to explain the data. It was only at this stage that themes were mapped back to review questions [17]. Finally, a thematic narrative synthesis was written for each review question checking back to the coded text to avoid de-contextualising data.

Rigour and reliability of the analysis were ensured in a number of ways. QSR NVIVO software was used for data management and to aid transparency of analysis. All studies were uploaded as pdf files to NVIVO. Inter-rater reliability was achieved by two primary reviewers [JW, KK] meeting to review codes and to check coded text throughout the process. A third reviewer [JS] independently read and made memos on a varied sample of studies, representing just under a third of studies included in the qualitative synthesis (*n* = 10 studies/11 papers). The reviewer then checked codes as displayed on NVIVO to ensure that there was consistency in the coding process between reviewers and between studies. A further means to build rigour was the use of a reflexive team blog and frequent meetings to discuss analysis. The authenticity of the final account was agreed by all reviewers [19].

## Results

In total, 15,230 records were identified through the database search with an additional 90 papers from other sources; of these 360 were eligible for second stage screening. Out of the 57 studies that were included in the overall systematic review [11],16 qualitative and 17 mixed methods studies were included in the qualitative synthesis (see Additional file 1: Table S1). Fourteen reported on UK-based peer interventions, eight were from US and eight from Canada. After thematic analysis, 18 organising codes and four thematic categories were mapped to the review question on positive and negative impacts (Table 2).

**Table 2:** Organising codes and thematic categories on positive and negative impacts

Thematic category	Organising code
Peer recruitment, training and support	Training and support mechanisms
	Recruitment and selection
	Retaining peer deliverers
	Payment and privileges
	Motivation for the role
Organisational support	Partnerships
	Institutional ‘buy-in’
	Funding and resource
Prisoner relationships	Providing practical support to prisoners
	Dependency
	Role tensions
	Awareness and utilisation
Prison life	Power and risk
	Contribution of peers to wider prison workforce and service delivery
	Impact on prison ethos and culture
	Peer interventions contributing to prison performance targets
	Integration of peer interventions into the prison
	Location of intervention
	Intervention arrangements and monitoring

Two thematic categories encompassed themes on the delivery of peer interventions; these were (1) peer recruitment, training & support and (2) organisational support. The other two categories encompassed themes relating to the social context of the prison; these were (3) prisoner relationships and (4) prison life (Table 2). Due to variation in the quality of data reported within the original studies, the results range from descriptive themes which lack depth through to cross cutting themes which are supported by rich data drawn from a number of studies. We indicate where there are strong or consistent data to support themes, or alternatively where there are particularly thin data.

### Peer recruitment, training and support

The delivery of peer interventions is dependent on the recruitment of prisoners, adequate training to prepare for the role and some ongoing supervision. Overall, there was a dearth of studies looking at these matters in depth. Recruitment methods were not reported in the majority of included studies, but there were consistent qualitative data on selection criteria for peer workers. Security factors were a major determinant of eligibility, with the exclusion of prisoners perceived to be at risk of security breaches, such as distribution of contraband [8, 20–23]. Other selection criteria included: providing a voluntary drugs test [20]; having knowledge of the system and ‘jail craft [21]; basic literacy skills [20]; and the period of time the prisoner was likely to be staying within the institution [20, 23, 24]. Interpersonal skills and commitment were considered in some interventions [8, 20, 25, 26], including the Listener scheme where a level of maturity was deemed necessary [21, 22]. Themes on the motivation of prisoners to take on a health role included an altruistic desire to support others [8, 20, 22], as well as personal benefits such as increased opportunity for parole [22, 25, 27], or being allocated a single cell [22]. Attrition and the difficulties retaining peer workers due to sudden movements of prisoners between institutions was a further cross cutting theme [8, 20, 23–25, 28–30].

The training of peer workers varied in content, duration, frequency and intensity across interventions, although there was very little qualitative evidence evaluating modes of delivery. One exception was the Canadian Peer Support Team programme that comprised 17 three-hour training sessions aimed at empowering women prisoners [27, 30]. Two further themes were the need for more comprehensive training in mental health issues [22, 25, 31] and the benefits of accredited training in providing prisoners with qualifications of use after release [8, 20, 25].

Supervision for peer workers was provided within interventions by prison staff through one-to-one or group meetings [8, 20, 25] or by external agencies [20, 21, 29]. There was little in depth evaluation of support systems, nevertheless most studies reported that prisoners valued support. Only one study reported inadequate support for participants in their peer role [32].

### Organisational support

A major theme was the importance of broader managerial support within the prison in order for schemes to operate successfully [8, 20–23, 26–28, 33]. Supportive relationships with other external agencies such as third sector organisations were also reported to be helpful [8, 21, 22]. Qualitative evidence revealed the importance of identified members of prison staff having responsibility for peer interventions as a mechanism to embed peer interventions within the prison [8, 23, 34]. The criticality of staff support at other levels within the prison, including assisting movement of prisoners around the institution, was also emphasised [20, 28]. Lack of funding and staff resources negatively impacted on staff support for peer interventions [24, 25].

### Prisoner relationships

Prisoner relationships covered themes on peer-to-peer interactions and on acceptability amongst the wider prison population. Recognition of the boundary between peer worker and recipients of the peer intervention was deemed important, with a number of studies reporting that peer workers knew when to ‘pass-on’ issues to healthcare professionals or prison staff [22, 27, 31, 33, 35]. There was some qualitative evidence of boundary issues occurring: for example, studies on the Listener scheme highlighted prisoner dependency on certain individuals [22] and peer workers’ concerns over maintaining appropriate boundaries for their role [36].

Quantitative studies in the main review showed that prisoners were generally satisfied with peer interventions [11]. The qualitative synthesis identified some reasons why prisoners did not utilise peer interventions, including: lack of awareness within the prisoner population [7, 23, 24, 30]; no personal need [23, 24]; concerns with confidentiality [23, 24, 27]; preference to discuss issues with trained staff, cell mates or family members [22, 24]; language barriers [23, 30]; and not demonstrating weakness to other prisoners [8, 22].

In terms of how peer interventions were seen by prison staff, lack of awareness and understanding of peer interventions was identified as a challenge [20, 24, 31, 33], but conversely regular communication [20, 33] and increasing familiarity of the intervention with time [37] were facilitating factors.

### Prison life

There were cross cutting themes on the place of peer interventions within prison life. Staff resistance was reported to be a significant barrier to the integration of peer interventions in prison settings [20, 21, 23, 30, 34, 37, 38], in some instances underpinned by security concerns [8]. Three studies reported that initial staff resistance receded as recognition of the value of peer-led services grew [20, 21, 37].

Placing prisoners in positions of relative power and trust meant that peer workers could become more susceptible to criticism and abuse from other prisoners by virtue of their alignment to staff [27]. At the same time, enhanced freedom and access to other prisoners could lead to increased security risks influencing how interventions were delivered [20, 31, 39]. Eleven studies described either perceived risks or actual instances where prisoners in peer roles had abused their position of trust [8, 20–25, 27, 30, 35, 39], with distribution of contraband such as tobacco or mobile telephones as the primary concern. More positively, peer workers were reported as acting as mediators between the prison population and staff, often creating more effective communication processes [23, 30, 31, 36].

There was qualitative evidence on the wider impacts on the prison system. Peer interventions could fill a gap in service provision in terms of helping prisoners with stress management and improving self-esteem [25]. In addition, they provided more fulfilling work opportunities within the prison setting, offering individuals the chance to gain skills and qualifications [8, 20, 25]. A cross cutting theme was the benefits of peer workers diverting demand from paid staff and thereby increasing staff availability for other duties [8, 20–23, 26, 30, 31, 40, 41]. Some qualitative evidence pointed to the positive impact on prison culture [8, 21–23, 27, 38, 42]. This ranged from peer workers being able to diffuse volatile situations and more cohesion between staff and prisoners to a more caring and humane atmosphere, reported in relation to US prison hospice schemes. In contrast, two studies concluded that peer interventions had very little impact on the prison regime [24, 25].

## Discussion

**Fig. 1:**
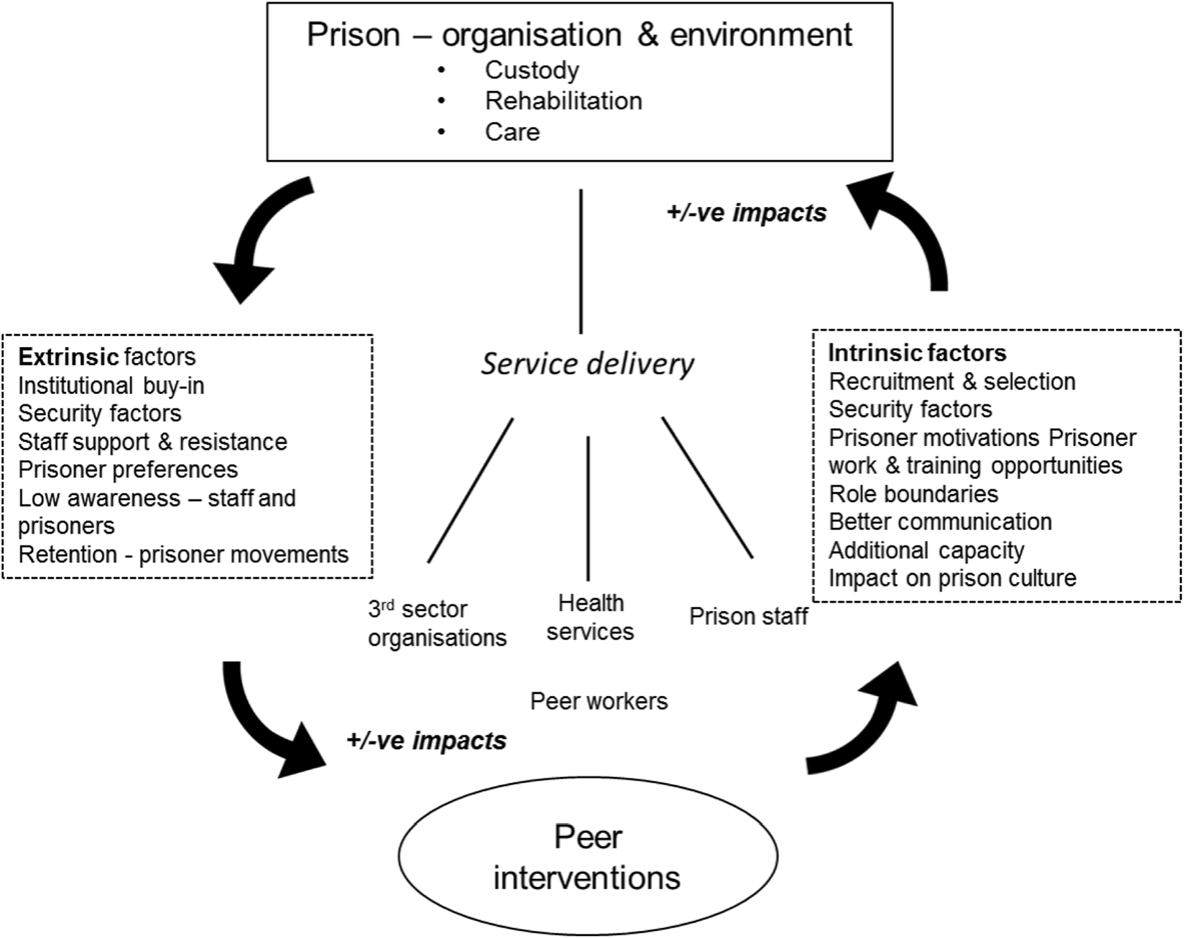
Factors related to the delivery of peer interventions in prison settings

In the prison setting, healthcare and preventive services are delivered within a wider system that is focused not primarily on health but on security and rehabilitation [2]. The results of this qualitative review confirm the assumption that there is an interplay between the prison system and peer interventions and this impacts on various stakeholder groups including prisoners, peer workers, prison staff, health services, and third sector organisations. A suite of factors were identified and these covered both *extrinsic* factors that modify the delivery of the peer intervention and factors *intrinsic* to peer interventions that impact on the wider organisation of the prison (see Fig. 1). This supports findings derived from expert evidence that contextual factors across organisational levels are critical to the success or otherwise of peer schemes [14]. The wider implications for service delivery are that prison health services need to adopt a settings approach that considers the whole environment [43] and to work in partnership with prison services to deliver peer interventions [8, 10].

The review has high relevance to UK health services, as the evidence came from mostly UK studies or from comparable schemes such as the Canadian Peer Support program [27]. One notable exception were the three US studies on peer volunteers in prison hospices [38, 42, 44, 45], which have low relevance are these relate to health systems where end-of-life care is organised differently. A further issue affecting applicability of review results is the relative lack of qualitative studies on peer interventions with women prisoners, exceptions being studies on the Canadian Support Program which was developed and delivered in women’s prisons [23, 24, 27] and a paper by Collica about building communities of support for women prisoners in New York State, US [29]. Across all studies in the review, there were limited qualitative data exploring the distinctive needs of women prisoners in comparison to men, or on gender issues more broadly. This would be an area worthy of further qualitative research as there may be differences between the development and delivery of peer interventions and wider support systems in men’s and women’s prisons.

Traditional systematic reviews of effectiveness risk a publication bias towards studies reporting positive outcomes. This qualitative review provides a useful counterbalance to the main review as negative impacts were identified, including additional demands and stress for staff and prisoners. These negative impacts should be considered in the light of strong evidence of positive mental health outcomes for prisoners who took on peer roles [12]. Health services have a duty of care to protect and promote the mental health of prisoners [1], including those who take on additional care responsibilities for others. Some schemes, such as Listeners, have well developed procedures for ongoing support and supervision [10]. Overall the results highlight gaps in knowledge about the processes associated with managing schemes well to protect both staff and prisoners.

Themes reflected an inherent tension between the goals of managing prison life, most notably maintaining security imperatives, and improving health [2, 6]. This has implications for the implementation of peer interventions as various aspects require active management; for example, selection procedures or working with prison staff to ensure prisoner movement for peer interactions. Security concerns about breaches of trust, both actual and perceived, was a major theme, but the literature indicates this is a complex issue, as peer interventions work on the basis of credibility and trust between peers [6, 29]. A prison peer worker therefore may have both the ability to communicate with other prisoners and concurrently pose a security risk. Some of the reasons for non-uptake, such as prisoner fears of breaches of confidentiality, demonstrate the importance of trust being built at different levels in order for peer schemes to be effective.

Peer interventions are best conceptualised as complex interventions taking place within a setting that has been described as a ‘total institution’ [46]. Many of the qualitative themes concerned relationships, both peer-to-peer and with staff. Organisational impacts were reported in terms of improvements to ethos and culture and management of staff workload. These wider impacts complement the individual level outcomes reported in the main effectiveness review [12]. In a period where investments need to be carefully justified, health programmes involving peer workers may add social value to the prison environment. More research is needed to investigate these organisational impacts from the perspective of different stakeholders. Some research pointed to importance of peer workers gaining qualifications and experience that may later assist rehabilitation [8, 20]. This side-effect from a health intervention is of value to the criminal justice system [7, 47] and has implications for commissioning [48]. Interventions that link health to desistance are of interest in UK policy [3], but more longitudinal research is needed to unpack the long term social and economic impacts.

There are limitations with both review methods and included studies which affect the strength of the conclusions. The choice of thematic analysis was made due to thin data and poor quality reporting in many studies. By including methodologically weak studies relating to a number of interventions, it was possible to identify a wide range of themes pertaining to prison life, but the strength of evidence is limited. Use of meta-ethnography [49] might have increased the strength of the conclusions, but only a small number of papers with thick descriptions of qualitative results could realistically have been included.

A transparent and rigorous analysis method [18] was used, with an additional process of quality review to check inter-rater reliability. Due to time constraints, and in line with the methods adopted [18], it was decided at protocol stage that only the abstract and findings sections would be included in the analysis. Given scientific reporting conventions, this should be sufficient, but some themes may have been missed if reported in discussion sections. While there were risks in synthesising results from heterogeneous studies, using NVIVO to label and retrieve full text helped avoid de-contextualising data.

The reflexive blog and team meetings helped ensure that authentic accounts were created [19], however it is possible that the team were sensitised to themes through other parts of the study, notably the expert symposium. Although qualitative research is not validated through numbers, it was concerning that many included papers reported data from very small samples; for example two or three participants [31, 35]. This reflects the constraints of undertaking research in a challenging environment, nevertheless it is difficult to be confident in those instances that data saturation has occurred. More high quality studies are needed that examine prisoner and staff perspectives using rigorous qualitative sampling and analysis methods.

## Conclusions

This review has identified influencing factors that shape the delivery of peer interventions within a prison setting, and how peer interventions can in turn affect prison life within that setting. The overall conclusion is that peer interventions to improve health cannot be considered stand-alone interventions. Health services therefore need to consider service delivery in terms of levels within the prison system from individual prisoner through to prison culture. Rather than a linear implementation process, the results suggest that a capacity building process is needed, both developing capacity in the offender population to provide advice, information and support, and in the organisation to enable smooth service delivery. This conclusion is in line with the tripartite agreement from national agencies in England, which emphasises the interdependence of health and justice services [3]. There is also potential to extend partnership working to draw in the expertise in the third sector in terms of volunteer management. Overall services need to actively address risks and mitigate negative factors in order to maximise added value of peer involvement in health service delivery.

## Additional file

Additional file 1: Table S1. Qualitative and mixed methods studies included in analysis for review question on positive and negative impacts (DOCX 34 kb)
