# Supplementary material for: A qualitative synthesis of the positive and negative impacts related to delivery of peer-based health interventions in prison settings
Source: BMC Health Serv Res. 2016 Sep 29;16:525. doi: 10.1186/s12913-016-1753-3 (PMC5041583; doi:10.1186/s12913-016-1753-3)
Supplement: Additional file 1: Table S1. — Qualitative and mixed methods studies included in analysis for review question on positive and negative impacts (DOCX 34 kb) [file 12913_2016_1753_MOESM1_ESM.docx]

**Table S1: Qualitative and mixed methods studies included in analysis for review question on positive and negative impacts**

| **Study** | **Country** | **Methods** | **Health topics** | **Nature of intervention/ scheme** | **Population/ setting** | **Validity**  **Score^[[1]](#footnote-1)^** |
| --- | --- | --- | --- | --- | --- | --- |
| Ashton C. Healing from the inside: an analysis of correctional service of Canada's circle of Knowledge Keepers Program. Canada: University of Toronto; 2010. | Canada | One semi-structured interview, one interview by email and publicly available documents including strategic plans, evaluation reports and operational outlines | Reduction of spread of infectious disease, specifically HIV/ AIDS and HCV | Circle of Knowledge Keepers (CKK) Peer education and Counseling (PEC) program: a peer educational and support programme, based within a ‘healing lodge’ | “Healing Lodge” – a small (28 bed) minimum/ medium security prison for Aboriginal women, incorporating Aboriginal healing practices, meaningfulness and cultural-connection. No fences, lock or bars. Women live in communal cottages, cook meals together. Most women are serving sentences of 3 years or less. | 3b |
| Betts-Symonds G. Community based health & first aid in action in Irish prisons. 3 year evaluation. Dublin: Irish Prison Service; 2012. | Ireland | Lessons learned workshop involving all prisons collectively.  Self reports from prisoner volunteers and staff.  Interviews with prison governors & staff | Health, hygiene and cleanliness | Peer education | 700 prisoners in Wheatfield prison, Dublin Ireland (medium-high security male prison) and their immediate family members | 3c |
| Blanchette, K. Eljdupovic-Guzina G. Results of a Pilot Study of the Peer Support Program for Women Offenders. Canada: Correctional Service of Canada; 1998. | Canada | Semi-structured interviews with offenders and staff.  Focus groups with offenders. Questionnaire surveys of offenders and staff. | General emotional/ mental health, psychological support and counselling | Peer support | Women resident in one of four small prisons in Canada: Nova Institution; Establishment Joliette; Grand Valley Institution; Edmonton Institution. | 2b |
| Boothby MRK. Insiders’ views of their role: toward their training. Can J Criminol 2011;53:424–48. | UK | Two focus groups; 3 Insiders participated. | General health/ support | Peer support | Male prison in the UK. The scheme supports prisoners who are new to the prison system. | 1a |
| Boyce I, Hunter G, Hough M. Peer advice project. An evaluation. London: St Giles Trust, King's College; 2009. | UK | 73 semi-structured interviews with 34 Peer Advisors based in prison and in the community.  64 semi-structured interviews with St Giles Trust staff, Prison Officers, resettlement, governors, probation and CARAT and other advice workers based in prison.  25 interviews with clients of the prison Peer Advice service.  4 focus groups with volunteers and clients of the community resettlement service, involving a total of 23 participants.  A focus group with clients of the prison Peer Advice service.  Observations of NVQ training and the delivery of Peer Advice work in prison.  Observations of mentoring work in the community.  Review of operational documents.  Review of project monitoring.  Collation of activity monitoring for prison and community projects. | Housing/ resettlement | Peer advisors | Serving prisoners in: 3 category B prisons (male), 1 Youth Offending Institution (male) | 2a |
| Brooker C, Sirdifield, C. New futures Health trainers: An impact assessment. Lincoln: University of Lincoln; 2007 | UK | Telephone interviews with 3 Health Trainer tutors and key staff; post-training survey (n=17).  Focus groups with Health Trainer tutors (n=11) and Health Trainers and attending a sample of steering group meetings (n=4) and obtaining minutes of these meetings.  Data captured through a ‘clinical activity data form’ and recorded information on the first fifteen clients that each Health Trainer saw. | Multiple health issues | Health Trainers | Serving prisoners in 4 adult prison, one Young Offenders Institution and one probation setting | 1a |
| Cichowlas JA, Chen Y-J. Volunteer prisoners provide hospice to dying inmates. Ann Health Law 2010;19:127–32. | USA | Volunteer inmates were interviewed | Social support | Peer support | Ill or dying prisoners in Dixon Hospice in Illinois. | 3c |
| Collica K. The prevalence of HIV peer programming in American prisons: an opportunity wasted. J Correct Health Care 2007;13:278–88. | USA | Quantitative and qualitative data collected via survey from 51 state departments of corrections and Federal Bureau of Corrections from January – September 2005. In total, 1,280 facilities and 1,427,279 inmates were covered by this survey. | HIV education | Peer education | All prisons and prisoners in USA | 3c |
| Collica K. Surviving incarceration: two prison-based peer programs build communities of support for female offenders. Deviant Behav 2010;31:314–47. | USA | One-to-one interviews with 49 female offenders (inmate peers) who work in a HIV prison-based peer program. | HIV/ AIDS and HCV (& other infectious diseases) | Peer education | Aimed at women in prison with HIV/AIDS.  One maximum and one medium security prison for women | 1b |
| Correctional Service of Canada. Opening the window on a very dark day: A program evaluation of the Peer Support Team in the Kingston Prison for women. Ottawa, Correctional Service of Canada; 2009 | Canada | Interviews with 12 peer counsellors (qualitative).  Survey of inmate population (quantitative). | Focus on women prisoners with a history of childhood sexual abuse. | Peer Support Team (peer counselling) | Women’s Prison in Kinston, Ontario (Canada). Women prisoners “in distress”. | 3b |
| Davies B. The Swansea Listener Scheme: views from the prison landings. Howard J Crim Justice 1994;33:125–35. | UK | In-depth interviews with 6 Listeners and 20 members of staff (selected randomly). 4 prisoners were based on the adult remand and convicted wings, two prisoners were located on the segregation unit. | Suicide/Self harm | Listeners | HMP Swansea (adult prison) | 2b |
| Delveaux K. Blanchette K. Results of an Evaluation of the Peer Support Program at Nova Institution for Women. Canada, Research Branch, Correctional Service of Canada; 2000. | Canada | Questionnaires, surveys of offenders and staff.  Semi-structured interviews with offenders and staff; focus groups with offenders. 18 offenders were interviewed; 1 peer support team (PST) member, 4 PST trainees and 13 other offenders. 22 staff members were interviewed. | General emotional/ mental health, psychological support and counselling | Peer support | Small women’s prison. Women prisoners, all serving sentences of two or more years and classified as minimum or medium security. | 3c |
| Dhaliwal R, Harrower J. Reducing prisoner vulnerability and providing a means of empowerment: evaluating the impact of a listener scheme on the listeners. Br J Forensic Pract 2009;11:35–43. | UK | Cross-sectional qualitative study. Qualitative interviews (intervention group only) using interpretative phenomenological analysis. 50-60 minute semi-structured interviews. Nine individuals met the inclusion criteria of having been a Listener for a minimum of six months, and 7 individuals agreed to take part | Suicide/Self harm | Listeners | Vulnerable or distressed prisoners, or those at risk of suicide. | 2b |
| Eamon KC, McLaren DL, Munchua MM, Tsutsumi LM. The peer support program at Edmonton Institution for Women. Forum 2012;11:28–30. | Canada | Quantitative (survey) and qualitative (interviews). Update to Blanchette 1998, carried out in April 1999. 26 prisoners and 13 staff members responded to survey. | General emotional/ mental health, psychological support and counselling | Peer Support | Edmonton Institution for Women. | 3b |
| Edgar K, Jacobson J, Biggar K.Time Well Spent: A practical guide to active citizenship and volunteering in prison. Prison Reform Trust London, Prison Reform Trust; 2011 | UK | Survey of prisons and interviews with prisoners and staff involved in active citizenship schemes in 12 prisons. (The report mainly draws on the qualitative data) | Multiple health issues | Peer support/ Listeners | 12 prisons. No information given on target recipients. | 2b |
| Foster J. Peer support in prison health care. An investigation into the Listening scheme in one adult male prison. London, School of Health & Social Care, University of Greenwich; 2011 | UK | Face-to-face interviews with 6 Listeners; 7 prisoners who had used the Listening scheme; 7 prisoners who had not used the Listening scheme; 2 prison officers; 6 health care professionals and the Samaritan Branch Prison Support Officer. Two Listener training sessions and one Safer Custody Group meeting were also observed. | Suicide/Self harm | Listeners | Adult category-B local male prison. Operational capacity 1103 | 1a |
| Hall B, Gabor P. Peer suicide prevention in a prison. Crisis 2004;25:19–26. | Canada | Data collected via examining records and documents including program manuals, minutes of meetings, training records and statistics from the Samaritans of Southern Alberta.  Interview and survey information also collected from key stakeholders including the SAMS in the Pen volunteers, correctional staff, general inmates, and professional staff.  17 interviews conducted with SAMS volunteers; 14 interviews conducted with professionals (parole officers); 12 interviews conducted with nurses, mental health staff, psychologists, chaplains, unit managers  Surveys completed by 126 inmates & 27 correctional officers. | Suicide prevention | Listeners | Medium security prison with capacity 585. Inmates have committed serious crimes.  Modal age category 18-29 years, followed by 30-39 years. Length of sentence ranged from 2 years to life. | 3c |
| Hoover J, Jurgens R. Harm reduction in prison: The Moldova Model. New York, Open Society Institute; 2009 | Moldova | Research involved 7 site visits to prisons and 1 to a pretrial detention facility as well as visits to the headquarters of the penitentiary system and NGO that provides harm reduction services in prisons. Interviews (numbers not known) were conducted with prisoners, pretrial detainees, NGO staff, penitentiary system officials & employees at both national & local levels. | HIV/ AIDS and HCV (& other infectious diseases) | Peer outreach | 7 prisons (6male prisons and 1 female prisons) | 3c |
| Hunter G, Boyce I. Preparing for Employment: Prisoners' Experience of Participating in a Prison Training Programme. The Howard Journal of Criminal Justice. 2009; 48:117-31 | UK | 44 semi-structured interviews with 28 offenders in prison and post-release. Interviewees were recruited from five prisons (n=22) and from the community project (n=56). | Housing/ resettlement | Peer advisors | Prisoners requiring housing advice in 5 prisons in South East England (Three Category B prisons (male), one young offender institution (male) and one female open prison.) | 1a |
| Jacobson, J, Edgar K. There when you need them most: Pact's first night in custody services. London, Prison Reform Trust; 2009. | UK | A study in England with 91 prisoners interviewed across 6 prisons- HMP Holloway, Wandsworth and Exeter and 3 comparator prisons (One-to-one interviews with 15 prisoners from each prison).  A study from a prison in Scotland (One-to-one interviews with 15 prisoners from HMP Edinburgh)/ | General health/ support | Peer support  The Prison Advice and care Trust (PACT) first night services, includes the use of insiders. PACT services include providing information about the prison; practical help such as making a phone call; emotional support; liaising with families | Prisoners on their first night in prison.  No differentiation between experienced prisoners (who had previously been in prison) and those who had never been in prison before. Holloway prison, Exeter prison, Wandsworth prison- intervention runs either in the Prison visitors centre or in dedicated first night suites. | 2c |
| Levenson J, Farrant F. Unlocking potential: active citizenship and volunteering by prisoners. Probat J 2002;49:195–204. | UK | Fieldwork was undertaken in eight prisons selected to represent all types of prisons, and which had also been identified from the survey as having examples of good practice. In total, 82 prisoners and 18 members of staff were spoken to. | Mental health, substance misuse, general health, housing support. | Peer support schemes, including Listeners. | National survey of all prisons in England and Wales. | 2b |
| Maull FW. Hospice care for prisoners: establishing an inmate-staffed hospice program in a prison medical facility. Hospice J 1991;7:43–55 | USA | Six month pilot program evaluation included: a review of all the visit reports submitted after each patient visit; all hospice team meeting minutes; all program correspondence; and the results of three separate anonymous questionnaires, one each for hospital staff (physicians, nurses, and correctional officers), patients, and inmate hospice volunteers. | General health/ support | Prison hospice volunteers | Ill prisoners at U.S. Medical Centre for Federal Prisoners in Springfield, Missouri | 2b |
| Munoz-Plaza CE, Strauss SM, Astone JM, Des Jarlais DC, Hagan H. Hepatitis C service delivery in prisons: peer education from the ‘guys in blue’. J Correct Health Care 2005;11:347–68. | USA | Inmates participated in individual face to face semi-structured interviews as well as a focus group. Drug treatment program staff members also participated in individual face to face interviews. Five inmates participated in the individual interviews and 6 in the focus group. | HIV/ AIDS and HCV (& other infectious diseases) | Peer education | A state correctional facility in California. Drug treatment program is located on a medium security prison yard that houses male inmates. Age range 20 – 50 years. | 2b |
| Penn State Erie. Final Report of the Process Evaluation of the Long Distance Dads Program. Erie. PA: Penn State Erie; 2001. | USA | This study had 4 components: survey of inmates; caregiver telephone interviews; face-to-face inmate interviews; and institutional data collection. Quantitative and qualitative data collected via survey from participants in 3, 12-week LDD sessions.  Telephone interviews were conducted with the caregivers of the LDD participants’ children. Face-to-face interviews were also conducted with a randomly selected sample of LDD participants from session 1, and also regularly collected DOC data. | Parenting | Peer-led fathering program. The LDD program is designed to assist incarcerated men in developing skills to become more involved and supportive fathers. | Pennsylvania Department of Corrections at the State Correctional Institution at Albion (Erie county). Fathers in prison. | 3c |
| Schinkel M, Whyte B. Routes out of prison using life coaches to assist resettlement. Howard J Crim Justice 2012;4:359–71. | UK | A total of 123 client/prisoner interviews were conducted; 49 prisoners pre-release, 54 post-release, and 20 at both stages. All of the Life Coaches (14) employed by the project were interviewed twice and consulted through focus groups, twice at different points in the life of the project. | Housing/resettlement | Peer mentoring | Based in Glasgow – prisons not stated. Prisoners serving sentences of between three months and four years. Service offered to eligible prisoners who are returning to Glasgow, Renfrewshire and North Lanarkshire. | 2b |
| Scott DP, Harzke AJ, Mizwa MB, Pugh M, Ross MW. Evaluation of an HIV peer education program in Texas prisons. J Correct Health Care 2004;10:151–73. | USA | Qualitative data collected via initial (March 1999) and follow-up (June/ July 1999) interviews with wardens and program coordinators.  Four initial (21 peer educators and 33 students) and follow-up focus groups (31 peer educators, 36 students) at each site with trained peer educators and their students. | HIV prevention | Peer education | Prisoners at 5 Texas prison facilities. A diversity of facilities was selected (small and large, short and long term, male and female prisoners) | 2c |
| Sirdifield C. Piloting a new role in mental health – prison based health trainers. J Ment Health Training Educ Pract 2006;1:15–22. | UK | Focus group discussions with 2 health trainers (opportunistic sample). | General health/ support | Health Trainer | All prisoners | 2b |
| Stewart W. Evaluating peer social care training in prisons. Prison Serv J 2011;195:43–6. | UK | Pre and post course questionnaires and semi-structured interviews with stakeholders | General health/ support | Peer support | 3 UK prisons.  Originally for older prisoners but to include those with learning disabilities, mental health problems and prisoners with physical and sensory disabilities. | 3c |
| Syed F, Blanchette K. Results of an Evaluation of the Peer Support Program at Grand Valley Institution for Women. Ottawa, ON: Correctional Service of Canada; 2000. | Canada | Interviews (21 prisoners: 4 PST members, 7 recipients, 10 non-recipients); 12 staff. | General emotional/ mental health, psychological support and counselling | Peer Support | Small women’s prison, n=78 at time of study. All were serving sentences of minimum 2 years and were rated at ‘minimum’ or ‘medium’ security levels. | 1c |
| Syed F, Blanchette K. Results of an Evaluation of the Peer Support Program at Joliette Institution for Women. Ottawa, ON: Correctional Service of Canada; 2000. | Canada | Semi-structured interviews with prisoners (n=29: 3 PST members, 7 recipients, 19 non-recipients) and staff (n=19) | General emotional/ mental health, psychological support and counselling. | Peer Support | Women’s prison in Canada. N=56 at time of study. All were serving sentences of minimum 2 years and were rated at ‘minimum’ or ‘medium’ security levels. | 2b |
| Taylor S. New South Wales Prison HIV Peer Education Program. Sydney: Department of Corrective Services Australia; 1994 | Australia | Pre (n=491)-post (n=458) questionnaire with prisoners.  ‘Random’ surveys conducted with staff and prisoners.  Review of the training and the programme materials | HIV prevention | Peer education | New South Wales Correctional Centres. 90% of inmates had been in other correctional centres. | 3b |
| The Learning Ladder Ltd. Mentoring for Progression: Peer Mentoring in a YOI. HMYOI Reading; 2010 | UK | Focus groups comprising of 3 mentors. | Enabling prisoners to reach “full potential” e.g. by gaining qualifications. | Peer mentoring | Reading YOI. Small prison for young offenders aged 18-21 years. | 3c |
| Wright KN, Bronstein L. Creating decent prisons: a serendipitous finding about prison hospice. J Offender Rehabil 2007;44:1–16.  Wright KN, Bronstein L. Organizational analysis of prison hospice. Prison J 2007;87:391–407. | USA | Telephone interviews/surveys- 17 questions lasting 45 mins on average. In total,14 respondents from the prison hospice unit; 5 social workers, 3 chaplains, 2 health administrators, 2 nurses, 1 clinical psychologist, 1 nonprofessional correctional staff member. | General health/ support | Prison hospice volunteers | Dying prisoners in 14 prison hospices in the USA | 2c |

1. Validity score: 1 = good internal validity, 2 = moderate internal validity and 3 = poor internal validity; a = highly relevant, b = of some relevance and c = not very relevant. [↑](#footnote-ref-1)
